# The extracellular matrix protein Edil3 stimulates osteoblast differentiation through the integrin α5β1/ERK/Runx2 pathway

**DOI:** 10.1371/journal.pone.0188749

**Published:** 2017-11-28

**Authors:** Sin-Hye Oh, Jung-Woo Kim, Yuri Kim, Mi Nam Lee, Min-Suk Kook, Eun Young Choi, Suhn-Young Im, Jeong-Tae Koh

**Affiliations:** 1 Department of Pharmacology and Dental Therapeutics, School of Dentistry, Chonnam National University, Gwangju, Republic of Korea; 2 Department of Biological Sciences, College of Natural Sciences, Chonnam National University, Gwangju, Republic of Korea; 3 Department of Oral and Maxillofacial Surgery, School of Dentistry, Chonnam National University, Gwangju, Republic of Korea; 4 Department of Biomedical Sciences, University of Ulsan College of Medicine, Seoul, Republic of Korea; University of Texas Southwestern Medical Center, UNITED STATES

## Abstract

Epidermal growth factor-like repeats and discoidin I-like domain 3 (Edil3) is an extracellular matrix protein containing an Arg-Gly-Asp (RGD) motif that binds integrin. Recently, Edil3 has been implicated in various biological processes, including angiogenesis and cellular differentiation. It can inhibit inflammatory bone destruction. The objective of this study was to explore the role of Edil3 in osteoblast differentiation and its underlying molecular mechanisms. In wild-type mice, high expression levels of Edil3 mRNA were observed in isolated calvaria and tibia/femur bones. Immunohistochemical analysis showed that Edil3 protein was localized along periosteum and calcified regions surrounding bone tissues. When murine calvaria-derived MC3T3-E1 cells were cultured in osteogenic medium containing 50 μg/ml ascorbic acid and 5 mM β-glycerophosphate, Edil3 mRNA and protein expression levels were increased. Treatment with Edil3 protein in growth media increased expression levels of alkaline phosphatase and osteocalcin gene and phosphorylation level of extracellular signal-regulated kinase (ERK). Edil3 treatment with osteogenic medium induced mineralization. Treatment with a neutralizing antibody against α5β1 and MEK inhibitor U0126 inhibited Edil3-enhanced osteogenic marker gene expression and mineral deposition. Edil3 increased protein expression levels of transcription factor runt-related transcription factor2 (Runx2). Edil3-induced Runx2 protein expression was suppressed by pretreatment with U0126. Taken together, these results suggest that Edil3 may stimulate osteoblast differentiation and matrix mineralization by increasing expression of Runx2 through α5β1 integrin /ERK pathway.

## Introduction

During bone development and remodeling, osteoblasts produce extracellular matrix (ECM) proteins which are regulators of matrix mineralization and under the control of transcription factors such as Runx2 [1]. ECM proteins such as collagen type I, fibronectin, and vitronectin mediate osteogenesis of mesenchymal stem cells through mineralization with high alkaline phosphatase (ALP) activity and up-regulated expression levels of bone sialoprotein (BSP) and osteocalcin (OC) gene [2].

In bone, ECM containing extracellular proteins play important roles in osteoblast cell proliferation, migration, and differentiation [3–5]. ECM proteins such as fibronectin, collagens, laminins, and vitronectin are able to interact with membrane integrins and initiate intracellular signals [6]. For instance, vitronectin and collagen type I can activate focal adhesion kinase (FAK)/paxillin and ERK/PI3K, respectively, to promote osteoblast differentiation by interacting with α2β1, α5β1, and αvβ3 integrins of mesenchymal stem cells (MSCs) [7]. Integrins expressed in osteoblasts are also involved in bone formation [8–10]. Previous studies have reported that integrin-ECM interaction can stimulate signaling mechanisms and promote early osteoblast-specific gene expression [8]. Notably, interactions among integrin, fibronectin, and collagen type I regulate osteoblast differentiation and cell fate *in vitro* [11, 12]. Several integrins such as α1β1, α2β1, α4β1, α5β1, αvβ3, and α11β1 are expressed in skeletal cells. They are known to have important roles in osteogenesis [13–15]. One of these integrins, α5β1, has been implicated in cell spreading, proliferation, differentiation, and survival. It also triggers osteoblast differentiation in MSCs [16, 17]. In addition, it has been demonstrated that interaction between integrin α5β1 and fibronectin is required for the ability of pre-osteoblasts to adhere to ECM and differentiate into mature osteoblasts [3, 11]. Osteogenesis is controlled by matrix proteins such as fibronectin that contains Arg-Gly-Asp (RGD) sequence, which can bind to integrin on the surface of osteoblasts, and trigger ERK and Rho signaling pathways [18–20]. Increasing integrin α5β1 expression in human MSCs results in activation of the FAK-ERK pathway and enhanced expression and activity of Runx2, leading to induction of osteogenic differentiation *in vitro* [19, 21–23]. Specifically, ECM/integrin interaction leads to activation of MAPK, ERK1 and ERK2, resulting in increased Runx2 phosphorylation and expression of osteoblast-specific genes [21–24].

Edil3, also known as Del-1, was first discovered as an ECM protein. It contains an RGD motif that can bind to integrin that is produced in the endothelium of vessels and by hypertrophic chondrocytes in developing embryos [25]. In addition, Edil3 has been described as an autocrine or paracrine secreted protein that participates in endothelial cell migration, angiogenesis, and branching morphogenesis of blood vessels [26, 27]. Recently, it has been demonstrated that Edil3 inhibits inflammation-induced bone destruction by regulating neutrophil recruitment and that Edil3 transgenic mice exhibit abnormal craniofacial development during embryonic growth [28, 29].

Expression and functional role of Edil3 in osteoblast function during osteogenesis remains unknown. Due to the important role of ECM-integrin interaction in osteoblast function, the objective of this study was to investigate the effect of Edil3 on osteoblast differentiation.

## Materials and methods

### Materials

Recombinant human Edil3 protein was purchased from R&D Systems (Minneapolis, MN, USA). Antibody against Col1a1 (Santa Cruz Biotechnology, Dallas, TX, USA) was used for immunostaining. Polyclonal antibodies against p-AKT, AKT, p-p38, p38, p-ERK, ERK, Runx2, and β–actin and inhibitors of MAPK (U0126, SB203580) and PI3K kinases (LY294002) were purchased from Cell Signaling Technology (Beverly, MA, USA). Antibody specific to Edil3 was supplied by Proteintech (Chicago, IL, USA). Antibodies specifically recognizing integrin αvβ5 and α5β1 were obtained from Millipore (Darmstadt, Germany). Anti-αvβ3, rat IgG, mouse IgG, anti-mouse IgG FITC, and anti-rat IgG FITC were purchased from eBioscience (San Diego, CA, USA).

### Tissue isolation

C57BL/6J mouse pups (Damool Science, Daejeon, Korea) at post-natal day 0 were sacrificed and tissues were isolated. Three pups were pooled for analysis of gene expression. All protocols were reviewed and approved by Animal Use and Care Committee of Chonnam National University.

### Immunohistochemistry

Immunohistochemistry was performed using VECTASTAIN ABC Kit (Vector Laboratories, Burlingame, CA, USA). Head containing calvaria was fixed with 4% paraformaldehyde solution overnight and then decalcified by Rapid Cal (BBC Coup, Stanwood, WA, USA) for 3 days at 4°C. The tissue was treated with ethanol dehydration, embedded in paraffin, and cut into 5 μm sections. Sections were then deparaffinized with xylenes and rehydrated with graded ethanol series. After washing three times with phosphate buffered saline (PBS) for 5 min each time, tissue sections were incubated with 3% hydrogen peroxide in methanol for 15 min. After washing twice with PBS, these sections were further incubated with anti-Edil3 primary antibody (1:100), anti-Runx2 primary antibody (1:50), or anti-Col1a1 primary antibody (1:50) overnight at 4°C in a moist chamber after blocking with 1% bovine serum albumin (BSA) for 10 min. After washing, biotinylated secondary antibody solution (VECTASTAIN ABC Kit) was incubated with the tissue for 10 min, these sections were washed with PBS and incubated with VECTASTAIN ABC reagent for 30 min at room temperature. Hematoxylin stain was then used for counter-staining. Immunostaining images were obtained using a DM microscope (Leica, Wetzlar, Germany) and an automatic digital slide scanner (Panoramic MIDI, 3DHISTECH Ltd, Hungary).

### Cell culture and osteoblast differentiation

MC3T3-E1, C3H10T1/2, and C2C12 cells were purchased from American Type Culture Collection (ATCC, Manassas, VA, USA). C2C12 and C3H10T1/2 cells were maintained in DMEM (Gibco, Grand Island, NY, USA) while MC3T3-E1 cells were cultured in α-minimal essential medium (α-MEM, Gibco) supplemented with 10% fetal bovine serum (FBS, Gibco). For differentiation experiments, MC3T3-E1 cells were seeded at density of 1×10^4^ cells/cm^2^ with growth media (GM, α-MEM containing 10% FBS). After 24 h of incubation, cells were exposed to indicated concentrations of Edil3 in GM or osteogenic media (OM) containing 50 μg/ml ascorbic acid and 5 mM β-glycerophosphate. As control for osteogenic differentiation, 200 ng/ml recombinant human bone morphogenetic protein 2 (BMP-2, Osstem implant, Seoul, Korea) in growth media was used for cell culture.

### Quantitative reverse transcription polymerase chain reaction (qRT-PCR)

Total RNA was prepared using TRIzol reagent (Invitrogen, CA, USA) according to the manufacturer’s instructions. cDNA was synthesized from total RNA using random primers (Promega, Fitchburg, WI, USA) and Moloney Murine Leukemia Virus reverse transcriptase (Promega). The reaction was performed at 42°C for 1 h followed by incubation at 94°C for 5 min in order to inactivate the reverse transcriptase. PCR reaction was performed in an Applied Biosystems Veriti 96-well fast thermal cycler (Thermo Fisher Scientific, Waltham, MA, USA) with the following program: incubation at 95°C for 10 min followed by 30 cycles of denaturation at 95°C for 30 sec, annealing at 55°C for 30 sec, extension at 72°C for 30 sec, and a final extension step at 72°C for 5 min. Amplified cDNA products were resolved in 1% agarose gel by electrophoresis. Real-time amplification of cDNA was conducted in StepOne Plus (Thermo Fisher Scientific) using Power SYBR Green PCR Master Mix (Thermo Fisher Scientific). PCR conditions were as follows: incubation at 95°C for 5 min followed by 40 cycles of denaturation at 95°C for 15 sec, annealing at 55°C for 15 sec, and extension at 72°C for 15 sec. The following primers were used: Edil3, forward (F) 5’-AAGGATTGGAAGCCCAGAGT-3’ and reverse (R) 5’-GCTCACAGCCAAGAAGTTCC-3’; Alp, (F) 5’-GATCATTCCCACGTTTTCAC-3’ and (R) 5’-TGCGGGCTTGTGGGACCTGC-3’; Oc, (F) 5’-CTCCTGAGTCTGACAAAGCCTT-3’ and (R) 5’-GCTGTGACATCCATTACTTGC-3’; Runx2, (F) 5’-GAGGGCACAAGTTCTATCTG-3’ and (R) 5’-CGCTCCGGCCCACAAATCTC-3’; β–actin, (F) 5′-TTCTTTGCAGCTCCTTCGTTGCCG-3′ and (R) 5′-TGGATGGCTACGTACATGGCTGGG-3′. Mean cycle threshold (Ct) values from triplicate measurements were employed to calculate gene expression. Expression levels of all mRNAs were normalized to those of endogenous β–actin.

### Western blotting

MC3T3-E1 cells were plated into 6-well plates at a density of 25,000 cells/well. After 24 h of incubation, cells were pre-treated with chemical inhibitors of MAPK and PI3K (10 μM U0126 for MEK/ERK, 10 μM SB203580 for p38, and 10 μM LY294002 for PI3K) for 1 h and then exposed to 200 ng/ml of Edil3 with fresh medium for designated time periods. Cells were then homogenized in cell lysis buffer (Cell Signaling). Protein concentration of cell lysate was quantified using a DC protein assay kit (Bio-Rad, Hercules, CA, USA). Equal amounts of protein were loaded onto 10–15% SDS-polyacrylamide gel and transferred electrophoretically to polyvinylidene difluoride membranes. These membranes were reacted with specific antibodies and visualized by enhanced chemiluminescence (Bio-Rad) according to the manufacturer’s recommendations using an LAS-4000 mini imager (Fujifilm, Tokyo, Japan). Densitometry analysis was performed using a Multi-Gauge analyzer (Fujifilm).

### Flow cytometry

To examine the expression of integrin αvβ3, αvβ5, and α5β1, flow cytometry analysis was performed for MC3T3-E1 cells using αvβ3, αvβ5, and α5β1 antibodies and anti-IgG FITC-conjugated antibodies. Briefly, 10,000 cells for each sample were mechanically collected, fixed in 4% paraformaldehyde for 15 min, and washed three times in PBS. These cells were reacted with primary antibodies against integrins followed by incubation with FITC-labeled secondary antibodies. Control samples were stained with secondary reagents only. Labeled cells were analyzed with a flow cytometer (Beckman Coulter, Paris, France).

### Alizarin red staining

Alizarin red (AR) staining was performed to evaluate the degree of matrix mineralization as described previously [30]. Briefly, cells were fixed with 70% ethanol and stained with 40 mM alizarin red solution (pH 4.2). These stained cells were photographed and 10% (w/v) cetylpyridinium chloride was then added to extract the stain. Absorbance of the extracted stain solution was measured at wavelength of 540 nm using a Multiskan GO (Thermo Fisher Scientific, Waltham, MA, USA).

### Luciferase assay

Cells were transfected with a Runx2 luciferase reporter plasmid containing six copies of Runx2-binding osteoblast specific element (6x OSE-Luc) and indicated plasmids (pEdil3, pRunx2, or empty vector as a mock control) using Lipofectamin2000 (Invitrogen). To measure luciferase activity, cells were harvested at 24 h post transfection and luciferase activity was measured using a luciferase reporter assay system (Promega). As an internal control, cytomegalovirus (CMV) β-galactosidase plasmid was co-transfected in each case. Luciferase activity was normalized to β-galactosidase activity.

### Statistical analysis

All experiments were repeated at least three times. Statistical analysis was performed using Student’s *t*-test or analysis of variance followed by Tukey’s multiple comparison test. Differences were considered significant at *p < 0*.*05*. Results are expressed as mean ± SEM for independent measurements performed in triplicates.

## Results

### Edil3 is expressed in bone tissues

**Fig 1:**
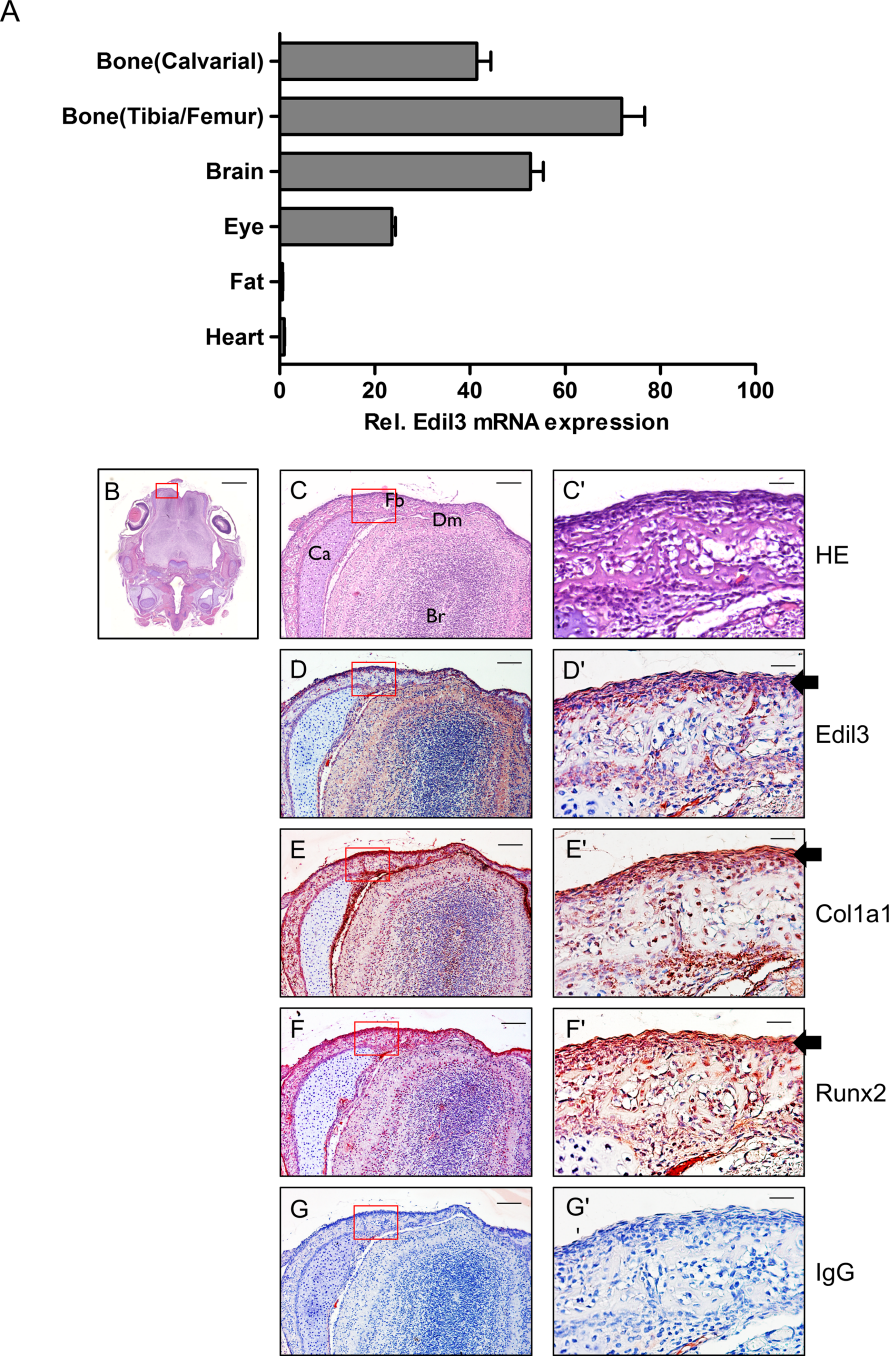
Expressions of Edil3 mRNA and protein in several organs and head tissues. (A) Edil3 mRNA levels in several tissues of mice at post-natal day (PN) 0 were assessed by qRT-PCR and normalized to β-actin levels. Edil3 level in the heart at PN 0 was adjusted to 1. All data are shown as mean ± S.E. (n = 3). (B) Hematoxylin and eosin (H&E) staining of coronal sections from newborn mouse heads. Scale bar indicates 1 mm. (C-G) High-magnification images for boxed area of B. Scale bar indicates 250 μm. (C'-G') Higher magnifications of boxed periosteal areas in the frontal bone of C-G. Scale bar indicates 50 μm. Specimens were stained with hematoxylin and eosin (B, C, and C’) or antibodies against Edil3 (D, D'), Col1a1 (E, E'), and Runx2 (F, F'). Anti-IgG antibody was used as a negative control (G, G'). Arrows indicate positive immunoreactivities against antibodies. Note that signals for Edil3, Col1a1, and Runx2 were strong in the periosteum. Br, Brain; Fb, frontal bone; Ca, Cartilages; Dm, Dura mater.

To examine tissue distribution of Edil3 expression, qRT-PCR was performed for several tissues collected from C57BL/6J mice at post-natal day 0. Edil3 mRNA was highly expressed in the calvaria, tibia/femur bones, brain, and eye compared to that in heart or fat tissues (Fig 1A). To further identify the expression of Edil3 protein in bone tissue *in vivo*, immunohistochemistry was performed using whole head sections of newborn mice. Consistent with the expression of major bone matrix protein Col1a1 protein, Edil3 protein expression was observed along periosteum and calcified regions surrounding bone tissues. Significantly high levels of Runx2 were closely approximated and overlapped with Edil3 expression in the periosteum (Fig 1B–1G’). These results suggest that extracellular matrix protein Edil3 could be produced by bone-forming periosteal cells and participate in cranial bone formation.

### Expression of Edil3 is increased during osteoblast differentiation

**Fig 2:**
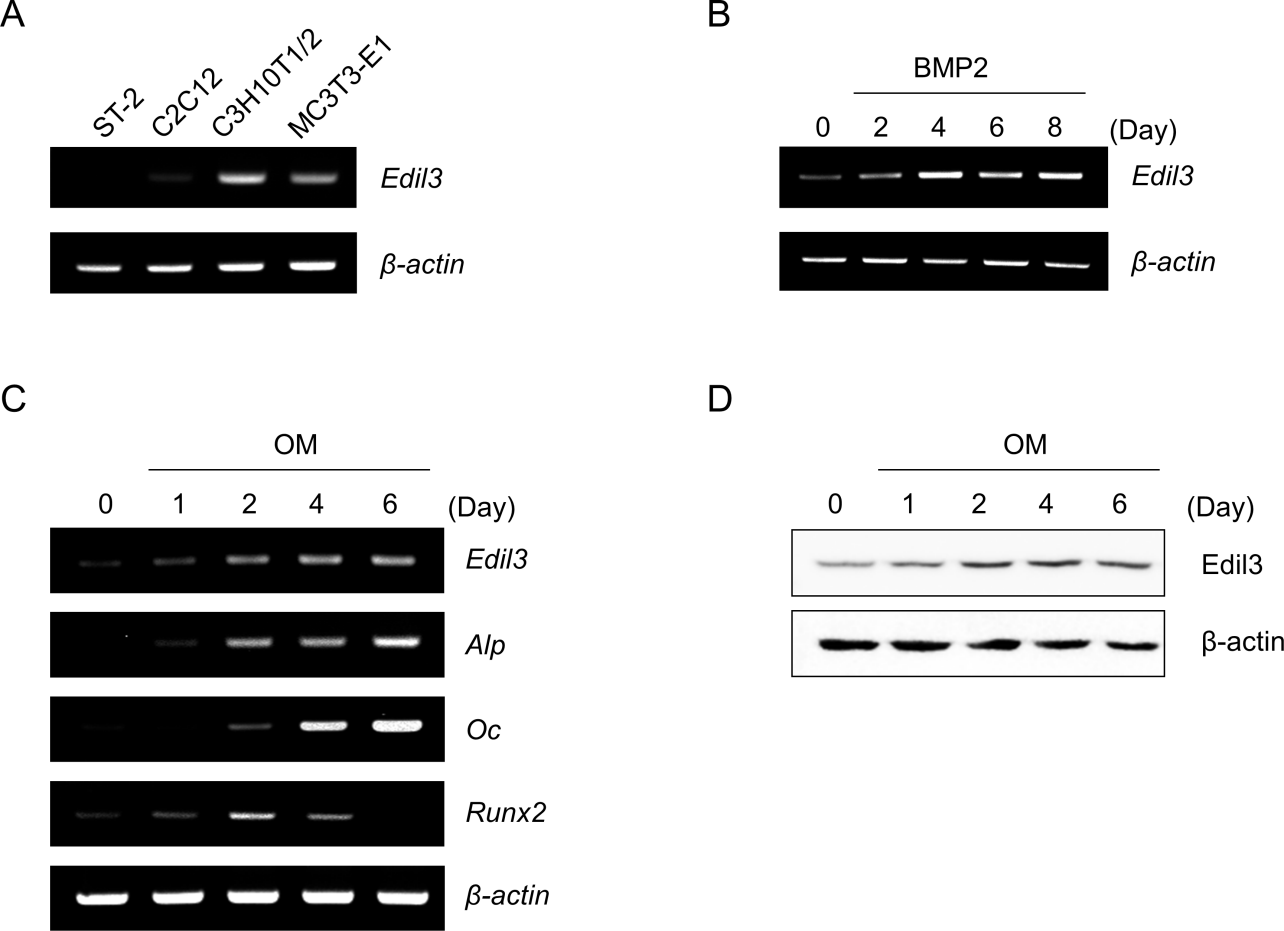
Expression of Edil3 mRNA and protein during osteoblast differentiation. (A) Endogenous expression of Edil3 mRNA in various progenitor cells. Total RNAs were isolated from cells that had been cultured for 3 days and used for RT-PCR with mouse Edil3 and β–actin primers. (B) Expression of Edil3 mRNA during BMP2-induced osteoblast differentiation. MC3T3-E1 cells were cultured with BMP2 (200 ng/ml) for up to 8 days. (C, D) MC3T3-E1 cells were maintained for up to 6 days in osteogenic medium (OM: α-MEM containing 10% FBS, 50 μg/ml ascorbic acid, and 5 mM β-glycerophosphate) and harvested at the indicated time points. RT-PCR was carried out using specific primers after total RNA isolation (B, C). Western blot analysis was performed with Edil3 and β-actin antibodies to evaluate Edil3 protein expression (D).

To explore the involvement of Edil3 in osteoblast differentiation, endogenous expression of Edil3 was examined in various osteoblastic lineage cells and differentiation-induced osteoblasts. Expression of Edil3 mRNA was highly detected in mesenchymal lineage C3H10T1/2 cells and calvaria-derived MC3T3-E1 cells (Fig 2A). Treatment with BMP2, a strong inducer of osteoblast differentiation, increased Edil3 mRNA expression up to 8 days (Fig 2B) in MC3T3-E1 cells. Treatment with osteogenic medium (OM), including ascorbic acid (50 μg/ml) and β-glycerophosphate (5 mM), also increased Edil3 mRNA expression with increased in osteoblast-specific gene expression such as Alp and Oc in MC3T3-E1 cells (Fig 2C). Runx2 mRNA expression levels were increased up to 2 days after the treatment. However, they were decreased to below control level at 6 days after the treatment. Edil3 protein level was also increased from day 2 up to day 6 in osteogenic medium-treated MC3T3-E1 cells (Fig 2D). These results suggest that Edil3 might be involved in osteoblast differentiation. Edil3 expression is gradually increased from early stage to late stage of differentiation.

### Treatment of Edil3 protein stimulates osteoblast differentiation

**Fig 3:**
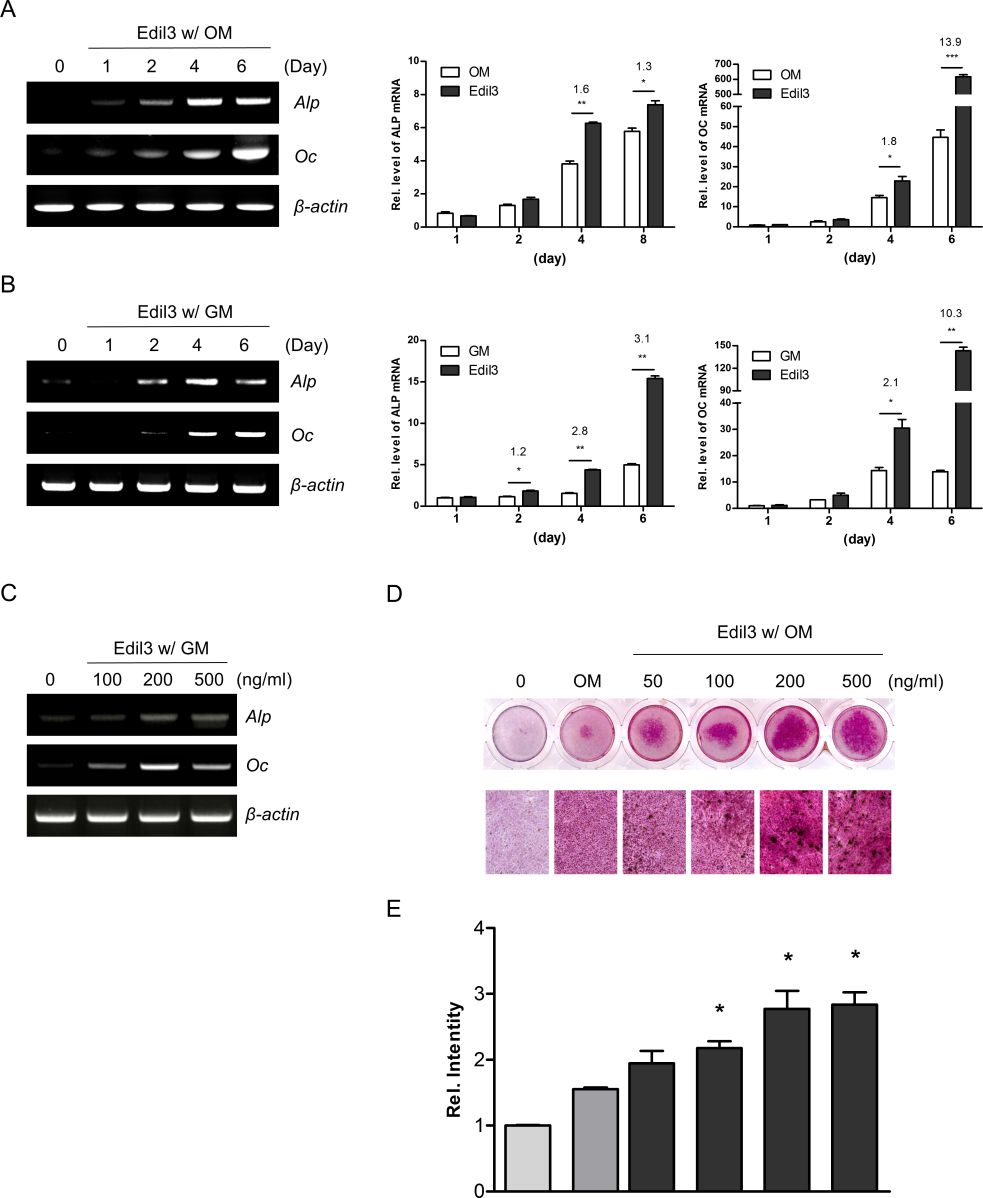
Effects of Edil3 protein treatment on osteoblast differentiation. (A, B) MC3T3-E1 cells were treated for indicated days with 200 ng/ml of Edil3 protein in the presence or absence of osteogenic medium. Cells were then harvested and RT-PCR (left panel) and qRT-PCR (middle and right panels) were performed using specific primers. The number indicates fold ratio between both groups. *, *P < 0*.*05;* **, *P < 0*.*01*. (C) Cells were treated with indicated concentration of Edil3 protein in growth medium (GM). After 4 days of treatment, cells were harvested and RT-PCR was performed. (D) Cells were cultured with indicated concentrations of Edil3 protein for 14 days in the presence of osteogenic medium and stained with alizarin red solution. (E) Graph showing quantified staining levels in (D). *, *P < 0*.*05* compared to OM-treated group.

To clarify the potential role of Edil3 in osteoblast differentiation, effect of exogenous Edil3 protein on osteogenic gene expression was evaluated in MC3T3-E1 cells. RT-PCR and qRT-PCR analyses consistently showed that treatment with recombinant Edil3 protein increased mRNA expression levels of Alp and Oc up to 6 days under both osteogenic and growth media condition (Fig 3A and 3B). In addition, Edil3 treatments dose-dependently increased mRNA expression levels of Alp and Oc under growth medium condition (Fig 3C). However, Edil3 treatment at concentration up to 500 ng/ml failed to affect the proliferation of MC3T3-E1 cells (S1 Fig).

Matrix mineralization, the most important phenomenon in bone formation, is regulated by various osteogenic factors [31]. To verify the functional role of Edil3 in matrix mineralization, alizarin red staining assay was performed for MC3T3-E1 cells cultured with Edil3 protein in mineralization medium. Treatment with Edil3 protein increased the formation of mineralized nodule in a dose-dependent manner (Fig 3D and 3E). Compared to RGD-containing fibronectin and osteopontin, it was similar (S2 Fig). These results suggest that Edil3 might be a stimulatory factor for osteoblast-specific gene expression and matrix mineralization.

### Edil3 induces osteoblast differentiation via interacting with α5β1

**Fig 4:**
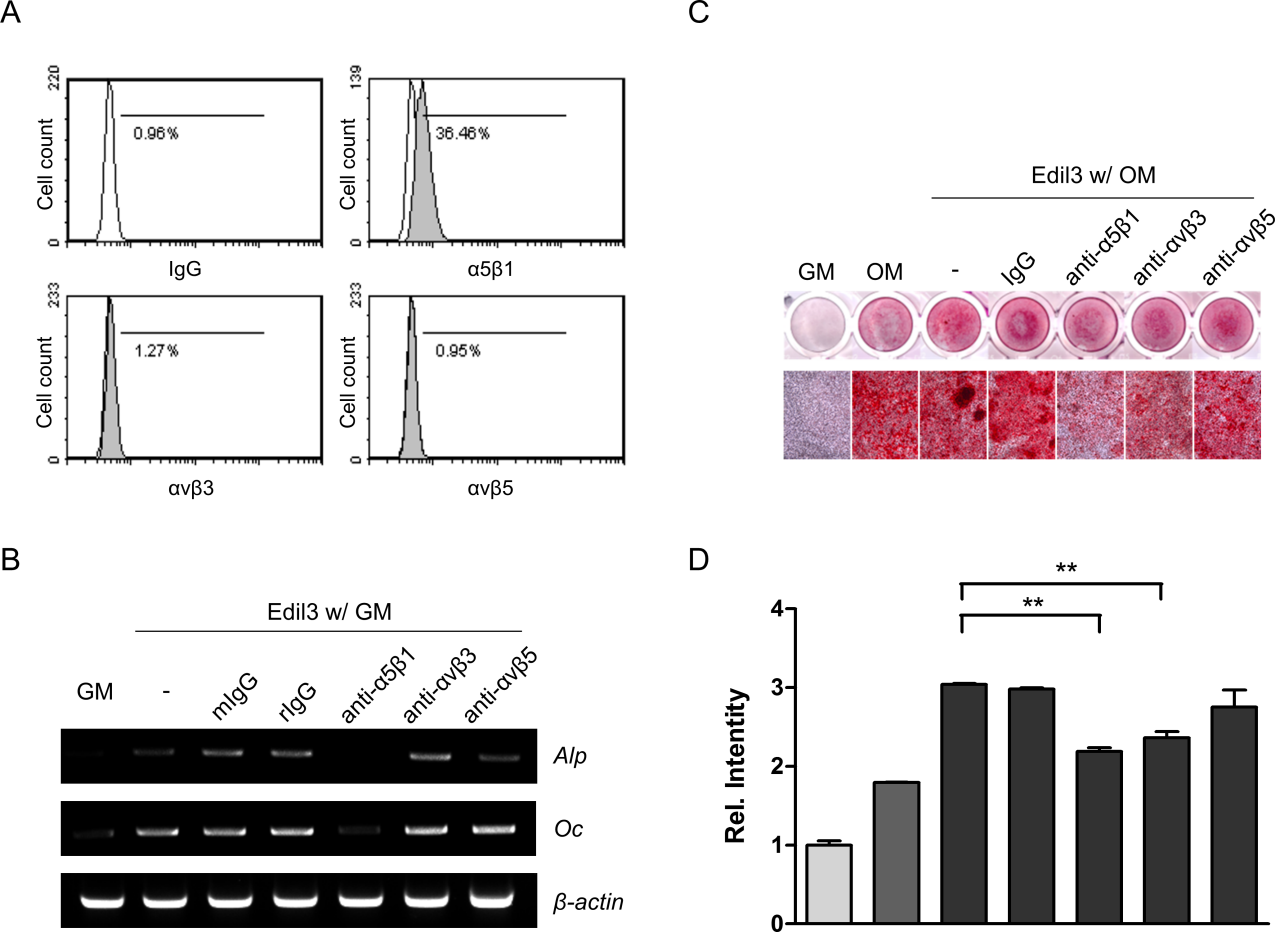
Effects of Edil3 on osteoblast differentiation following treatment with integrin antibodies. (A) Integrin expression in MC3T3-E1 cells. Flow cytometry analysis revealed that the distribution of cells stained for integrins (shaded regions) and IgG as background control (open regions). (B) Effects of integrin-blocking antibodies on Edil3-induced osteoblast differentiation. Cells were incubated with 1 μg/ml of specific or control antibodies and 200 ng/ml of Edil3 for 2 days, and then harvested for RT-PCR analysis. (C) MC3T3-E1 cells were treated with Edil3 and indicated antibodies against integrins, and maintained for 14 days in osteogenic medium. These cells were stained with alizarin red solution. (D) Graph showing quantification of staining levels in (C). **, *P < 0*.*01* vs. indicated group. mIgG, mouse IgG; rIgG, rat IgG.

Edil3 contains an RGD motif in the EGF-like domain. It contributes to cell adhesion, migration, and angiogenesis by binding to αvβ3 and αvβ5 integrins [32]. Many matrix proteins regulate osteoblast differentiation via interacting with integrins [8, 11]. In the present study, we performed flow cytometry analysis to assess whether MC3T3-E1 cells grown in normal growth medium might express different integrins. As shown in Fig 4A, expression of α5β1, αvβ3, and αvβ5 was detected. However, expression of α5β1 was the most prominent compared to that of αvβ3 or αvβ5. To identify the involvement of specific integrins in Edil3-induced osteogenic gene expression and matrix deposition, cells were pre-treated with antibodies against specific integrins to inhibit ligands of integrin. Treatment with an antibody against α5β1 inhibited Edil3-induced Alp and Oc expression (Fig 4B). Formation of mineralized nodules by Edil3 was also decreased in the group pretreated with an antibody against α5β1 (Fig 4C and 4D). These results indicate that integrin α5β1 mediates Edil3-induced osteoblast differentiation and mineralization.

### ERK signaling pathway mediates Edil3-induced osteoblast differentiation

**Fig 5:**
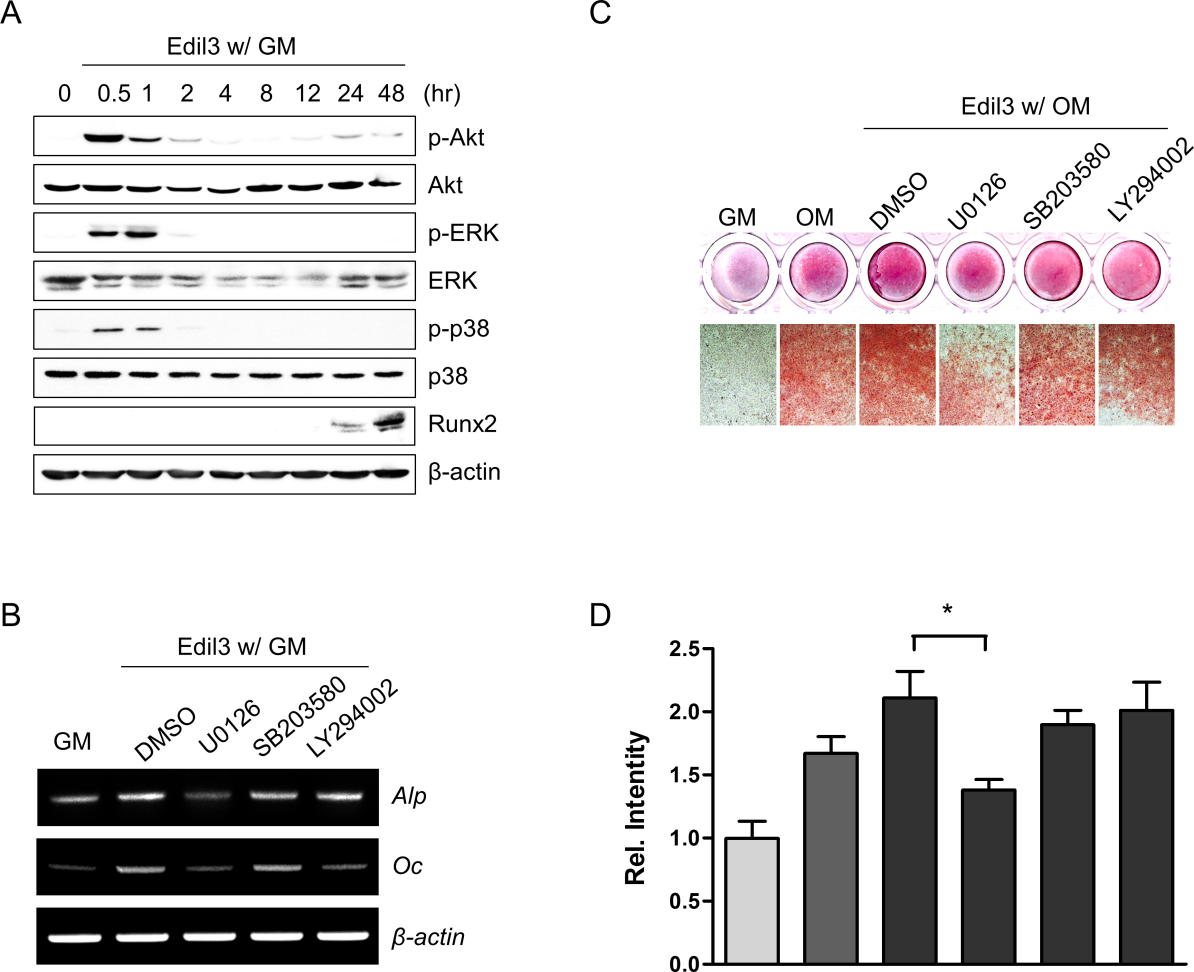
Involvement of ERK signaling in Edil3-enhanced osteoblast differentiation. (A) MC3T3-E1 cells were treated with 200 ng/ml of Edil3 for the indicated time period. Immunoblot analysis was performed with specific antibodies against Akt, ERK, p38, and Runx2 in order to identify its signaling pathway. (B) Cells were cultured with specific inhibitors (MEK/ERK inhibitor U0126, 10 μM; p38 inhibitor SB203580, 10 μM; or Akt inhibitor LY294002, 10 μM) for 1 h, separately, and then treated with 200 ng/ml of Edil3. These cells were harvested after 4 days of treatment and subjected to RT-PCR. (C) Cells were treated with U0126, SB203580, or LY294002 for 1 h. After removing inhibitor-containing medium, cells were incubated with 200 ng/ml Edil3 in osteogenic medium. After 14 days, cells were harvested for AR-staining. (D) Graph showing quantification of staining levels in (C). *, *P < 0*.*05* vs. indicated group.

Integrin and MAP kinase signaling pathways are involved in ECM-related osteoblast differentiation [21, 23]. To elucidate the underlying mechanism of Edil3-induced osteoblast differentiation, phosphorylation levels of PI3K-Akt and MAP kinase (ERK and p38) were determined by Western blotting in MC3T3-E1 cells. Treatment of Edil3 with growth medium increased phosphorylation levels of Akt, ERK, and p38 peaking at 0.5–1 h after treatment (Fig 5A). Phosphorylation levels in Edil3-treated group were greater than those in matched time-control group without Edil3 treatment (S3 Fig). On the other hand, Runx2 protein expression was increased at 24 h after Edil3 treatment (Fig 5A). Selective MEK/ERK inhibitor U0126 inhibited Edil3-induced Alp and Oc mRNA expression under growth medium condition (Fig 5B). In addition, the selective MEK/ERK inhibitor U0126 significantly inhibited Edil3-induced matrix mineralization under osteogenic medium (Fig 5C and 5D). These results suggest that Edil3 can stimulate osteoblast differentiation through ERK pathway, at least in part.

### Edil3 regulates Runx2 protein expression through ERK pathway

**Fig 6:**
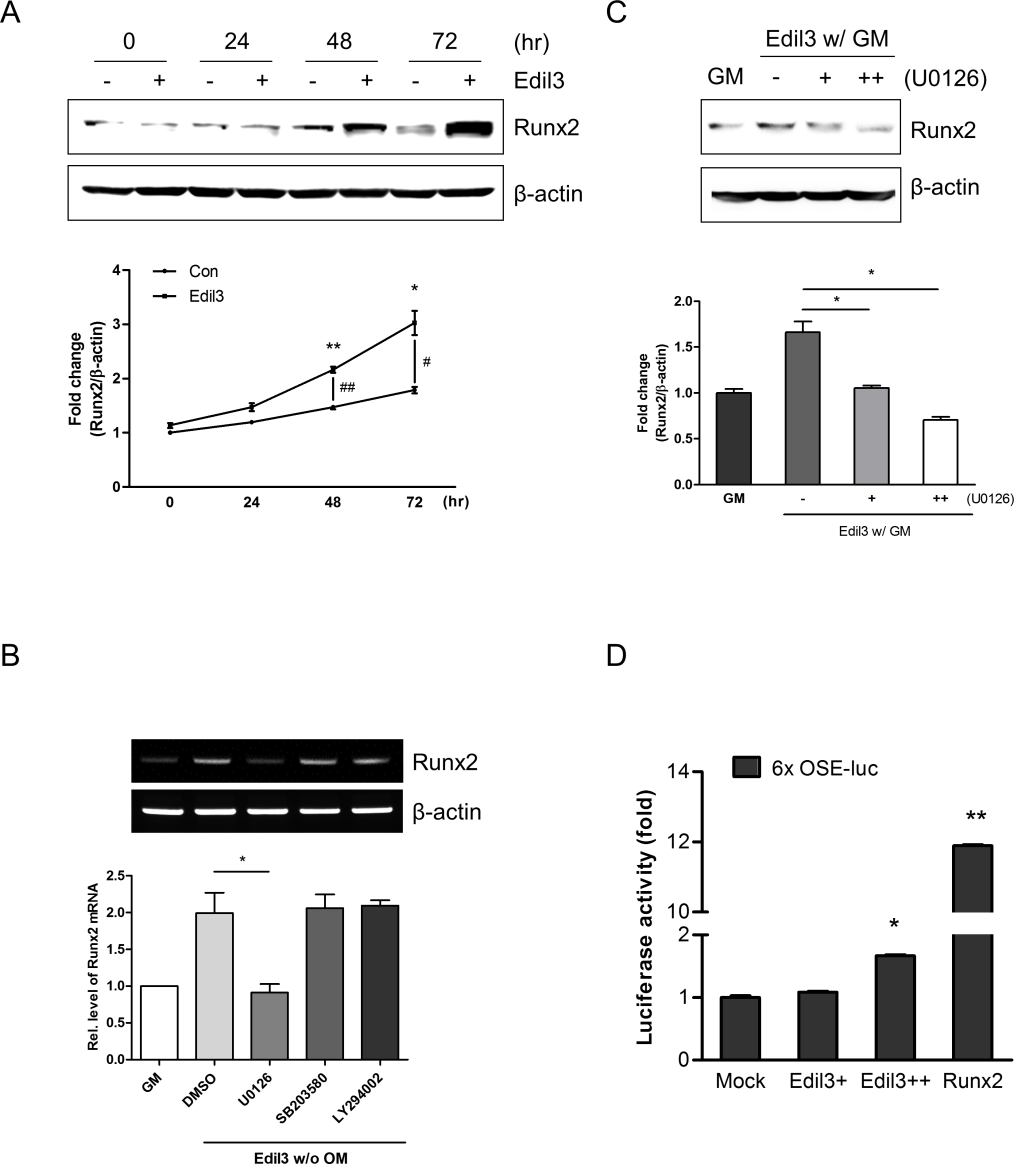
Effects of Edil3 treatment on Runx2 expression and transcriptional activity. (A) Runx2 protein expression. MC3T3-E1 cells were treated with or without 200 ng/ml of Edil3 for indicated time periods and then subjected to Western blot analysis. Upper panel is a representative image. Lower graph shows quantitative and relative level of Runx2 protein (n = 3). *, *P < 0*.*05*; **, *P < 0*.*01* vs. before treatment. ^#^, *P < 0*.*05*; ^##^, *P < 0*.*01* vs. time control group. (B) Cells were cultured with specific inhibitors (MEK/ERK inhibitor U0126, 10 μM; p38 inhibitor SB203580, 10 μM; or Akt inhibitor LY294002, 10 μM) for 1 h followed by treatment with 200 ng/ml of Edil3 for 4 days. Cells were then harvested for RT-PCR. (C) Cells were treated with Edil3 in the presence or absence of U0126 (+, 5 μM; ++, 10 μM) for 1 h. After changing into growth medium containing 200 ng/ml of Edil3, cells were cultured for 48 h. Western blot analysis was performed with indicated antibodies to evaluate Runx2 expression. Upper panel is a representative image and lower graph shows quantitative level of Runx2 protein (n = 3). *, *P < 0*.*05* vs. indicated group. (D) Effects of Edil3 on luciferase activity using a Runx2 luciferase reporter plasmid containing six copies of Runx2-binding osteoblast specific element (6x OSE-Luc). MC3T3-E1 cells were co-transfected with indicated plasmids, including 6x OSE-luc (500 ng), pYX-Asc-Edil3 (+, 300 ng; ++, 600 ng), pCS-Myc-Runx2 (600 ng), and a control mock plasmid (600 ng) with pCMV-β-galactosidase (500 ng). Luciferase activity was measured and normalized to β-galactosidase activity. *, *P < 0*.*05*; **, *P < 0*.*01* vs. Mock control.

Roles of MAPK and Runx2 in osteogenic differentiation have been widely recognized. To explore whether Edil3 might regulate osteogenic differentiation via the ERK-Runx2 pathways, Western blot and qRT-PCR analyses were performed. Treatment with Edil3 in growth medium increased Runx2 protein expression in a time-dependent manner (Figs 5A and 6A). Runx2 expression in Edil3-treated group was significantly higher than that in untreated control group at each time-point (Fig 6A). Pretreatment with MEK/ERK inhibitor U0126 prominently suppressed Edil3-induced Runx2 mRNA expression compared to chemical inhibitors of p38 and PI3K (Fig 6B). In addition, pretreatment with U0126 dose-dependently inhibited Edil3-induced Runx2 protein expression (Fig 6C).

To further examine whether Edil3 treatment might affect Runx2 transcriptional activity, luciferase assay and Western blot analysis were performed. When MC3T3-E1 cells were co-transfected with Edil3 expression plasmid and Runx2-binding 6X OSE-Luc reporter, luciferase activity was increased significantly (Fig 6D). These results suggest that Edil3 can increase osteoblast differentiation through enhancing Runx2 expression via activating ERK pathway.

## Discussion

This study demonstrated that treatment with extracellular matrix protein Edil3 stimulated *in vitro* osteoblast differentiation through activating integrin α5β1-ERK pathway and upregulating Runx2 expression. Our results also showed that Edil3 was expressed in bone tissue and its protein levels were increased during *in vitro* osteoblast differentiation.

Bone tissue consists of many extracellular matrix proteins, including collagen, fibronectin, laminin, bone sialoprotein, and osteocalcin that play important roles in osteoblast differentiation, proliferation, and survival [33]. These matrix proteins produced from bone-forming osteoblasts can act as autocrine regulators for bone formation [34]. Edil3 was firstly characterized as an ECM protein secreted from endothelial cells that could stimulate angiogenesis [26, 35]. In the present study, Edil3 mRNA and protein expression were observed in bone tissues and osteoblast-lineage cells. Its expression was found to be increased during osteoblast differentiation of MC3T3-E1 cells (Figs 1 and 2). Immunohistochemical analysis showed that Edil3 protein was expressed in periosteal tissue containing osteoblasts and mineralized regions. Furthermore, localization of Edil3 was found to be similar to that of Col1a1 (Fig 1). Based on these findings, we cautiously hypothesize that Edil3 represents a bone matrix protein that plays a role in osteoblast differentiation and mineralization.

Integrin receptors and MAPK pathways are involved in ECM-mediated osteoblast differentiation [24]. For example, type I collagen interacts with β1 integrin of osteoblasts and leads to activation of MAPK pathways and Runx2 phosphorylation, thereby stimulating osteoblast differentiation via upregulation of the osteoblast transcriptional program, including expression of osteocalcin [36]. Fibronectin, a natural ligand for several integrins including α5β1 and α4β1, enhances *in vitro* vascular calcification by promoting osteoblastic differentiation of vascular smooth muscle cells via ERK pathway and Runx2 [37, 38]. The present study revealed that matrix protein Edil3 could also enhance osteoblast-specific gene expression and matrix mineralization via interaction with integrin α5β1, activating ERK pathway and increasing Runx2 expression. Even in growth medium condition, treatment with Edil3 increased Alp and Oc mRNA expression (Fig 3B), suggesting that Edil3 can compensate for the lack of ECM/integrin signaling. In addition, Edil3 protein under osteogenic medium appeared to have effect on mineralization similar to fibronectin and osteopontin (S2 Fig). The mode of action of Edil3 via the integrin-MAPK pathway is similar to that of type I collagen and fibronectin, although the mechanism involved in this process has not been defined yet.

Several studies have shown that Edil3 is a ligand for αvβ3 and αvβ5 integrins, especially in endothelial cells in developing embryos. Integrin expression on cell surface can vary depending on culture conditions and contact surface [39]. In the present study, prominent expression of α5β1 was observed in MC3T3-E1 cells. Treatment with a blocking antibody for integrin α5β1 inhibited Edil3-induced mineralization and osteoblast-specific gene expression. Our results suggest that Edil3 may act as a ligand for integrin α5β1 receptor in osteoblasts, and has a potential role in osteoblast differentiation and possibly mineralization. In the present study, treatment with anti-αvβ3 antibody in osteogenic medium inhibited Edil3-induced mineralization. However, treatment with anti-αvβ3 antibody in growth medium did not affect the expression of Alp or Oc mRNA (Fig 4B and 4C). Such discrepancy in antibody effects on gene expression and mineralization might be due to different culture condition. Considering these results, αvβ3 might be involved in OM-induced mineralization in an Edil3 independent manner.

Extracellular stimuli such as mechanical force and ECMs can enhance osteoblast differentiation with increased ERK phosphorylation and Runx2 activation or expression [22, 40]. ERK-mediated Runx2 activation for the differentiation process has been well defined. However, kinase induction of Runx2 expression has not been clarified yet. A previous study has shown that continuous mechanical stress can stimulate osteogenic differentiation with increases in phosphorylation level of ERK1/2 and Runx2 expression and that treatment with ERK1/2 selective inhibitor (U0126) clearly downregulates Runx2 expression as well as gene expression levels of other osteogenic-related factors in bone marrow stem cells [40]. Runx2 P1 promoter contains Y-repeated elements which bind to transcriptional factor Sp1 and ELK1. ERK recognizes ELK1 protein by binding to its D-domain and FxF motif and phosphorylates its intervening phospho-acceptor motifs [41]. Overexpression of ELK1 upregulates Runx2 mRNA and protein expression levels with increases in phosphorylation of ELK1 [42. 43]. In this study, Edil3 increased Runx2 expression with increased ERK1/2 phosphorylation. U0126 also inhibited Edil3-mediated Runx2 expression. Based on the findings, we putatively interpret that Edil3 can stimulate Runx2 expression and osteoblast differentiation via ERK1/2 pathway. Although we do not understand exactly how ERK activation can increase Runx2 expression, presently we assume a possibility that transcription factor ELK1 targeting Runx2 gene might be involved in Edil3 mediated Runx2 expression because Edil3 can induce ERK1/2 phosphorylation. It might subsequently stimulate ELK1 to activate Runx2 transcription over a few days. Further studies are needed to define the exact mechanism involved.

Many ECM proteins take part in bone formation via regulating migration and adhesion of osteoblast-lineage cells, angiogenesis, and differentiation [2, 8]. In the present study, we observed that Edil3 (0–500 ng/ml) did not affect proliferation of MC3T3-E1 cells for up to 48 h (S1 Fig). Edil3-induced osteoblast differentiation and mineralization in the context of cell adhesion, migration, apoptosis, and so forth, further studies are still needed.

In summary, Edil3 can stimulate osteoblast differentiation via inducing integrin α5β1 signaling-mediated ERK-dependent Runx2 expression, leading to increased expression of Alp and Oc. This study provides a mechanistic explanation for the role of Edil3 in osteoblast differentiation through α5β1/ERK/Runx2 pathway, suggesting that Edil3 might be useful for enhancing mineralization. Based on these findings, Edil3 could be used for surface modification of biomaterials and therapeutic targets in bone tissue engineering applications.

## Supporting information

S1 Fig Effects of Edil3 treatment on cell proliferation. MC3T3-E1 cells were cultured with recombinant Edil3 protein at designated concentration in growth medium. After 24 h or 48 h of incubation, cell proliferation was detected by BrdU assay kit (Cell signaling Technology).
(TIF)

S2 Fig Effects of ECM protein Edil3, fibronectin, and osteopontin on matrix mineralization. MC3T3-E1 cells were treated with fibronectin (FN, 200 ng/ml, Sigma-Aldrich), osteopontin (OPN, 200 ng/ml, R&D Systems), or Edil3 (200 ng/ml, R&D Systems) separately and maintained in osteogenic medium containing 50 μg/ml ascorbic acid and 5 mM β-glycerophosphate. After 14 days of culture, cells were harvested and stained with alizarin red solution.
(TIF)

S3 Fig Effects of Edil3 on phosphorylation of Akt, ERK, and p38. MC3T3-E1 cells were incubated with growth medium for the indicated time period in the absence or presence of Edil3 (200 ng/ml). Cells were harvested and immunoblot analysis was performed using specific antibodies against p-Akt, p-ERK, p-p38, and β-actin. Note that Edil3 treatment increased phosphorylation of these kinases compared to the time-course control without Edil3.
(TIF)
