# Supplementary figures and images for: The extracellular matrix protein Edil3 stimulates osteoblast differentiation through the integrin α5β1/ERK/Runx2 pathway
Source: PLoS One. 2017 Nov 28;12(11):e0188749. doi: 10.1371/journal.pone.0188749 (PMC5705136; doi:10.1371/journal.pone.0188749)

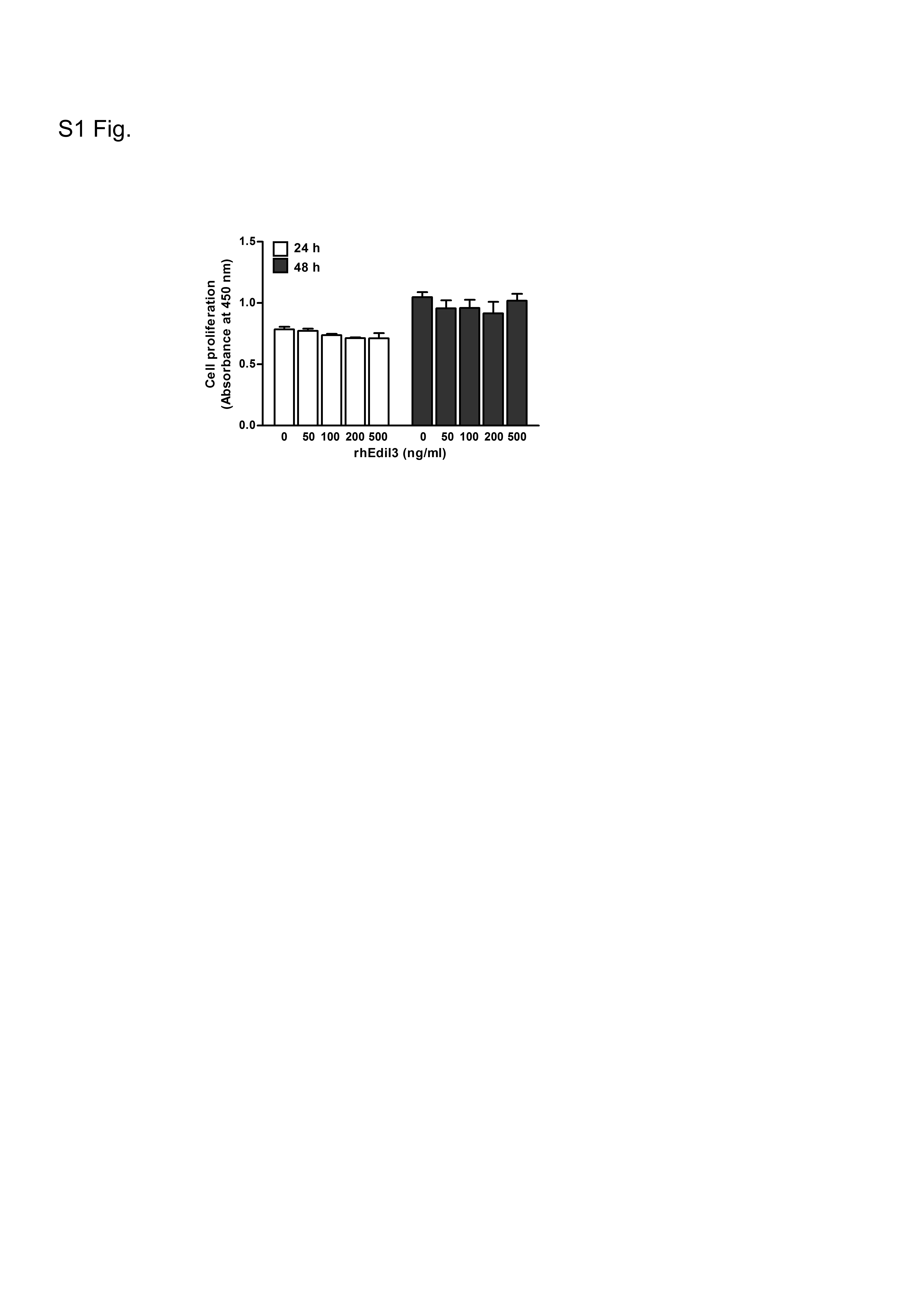

Supplement: S1 Fig — MC3T3-E1 cells were cultured with recombinant Edil3 protein at designated concentration in growth medium. After 24 h or 48 h of incubation, cell proliferation was detected by BrdU assay kit (Cell signaling Technology). (TIF) [file pone.0188749.s001.TIF]

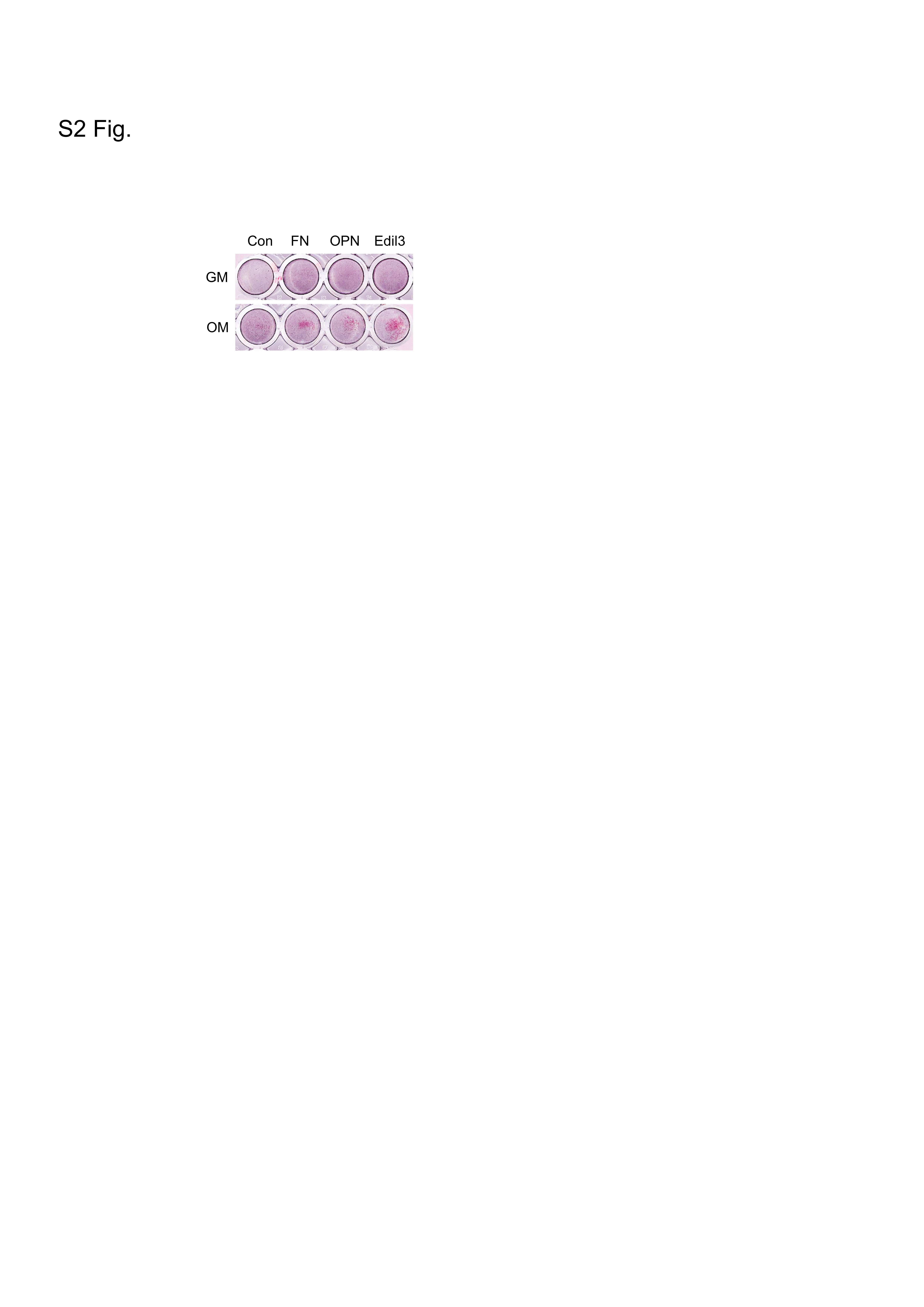

Supplement: S2 Fig — MC3T3-E1 cells were treated with fibronectin (FN, 200 ng/ml, Sigma-Aldrich), osteopontin (OPN, 200 ng/ml, R&D Systems), or Edil3 (200 ng/ml, R&D Systems) separately and maintained in osteogenic medium containing 50 μg/ml ascorbic acid and 5 mM β-glycerophosphate. After 14 days of culture, cells were harvested and stained with alizarin red solution. (TIF) [file pone.0188749.s002.TIF]

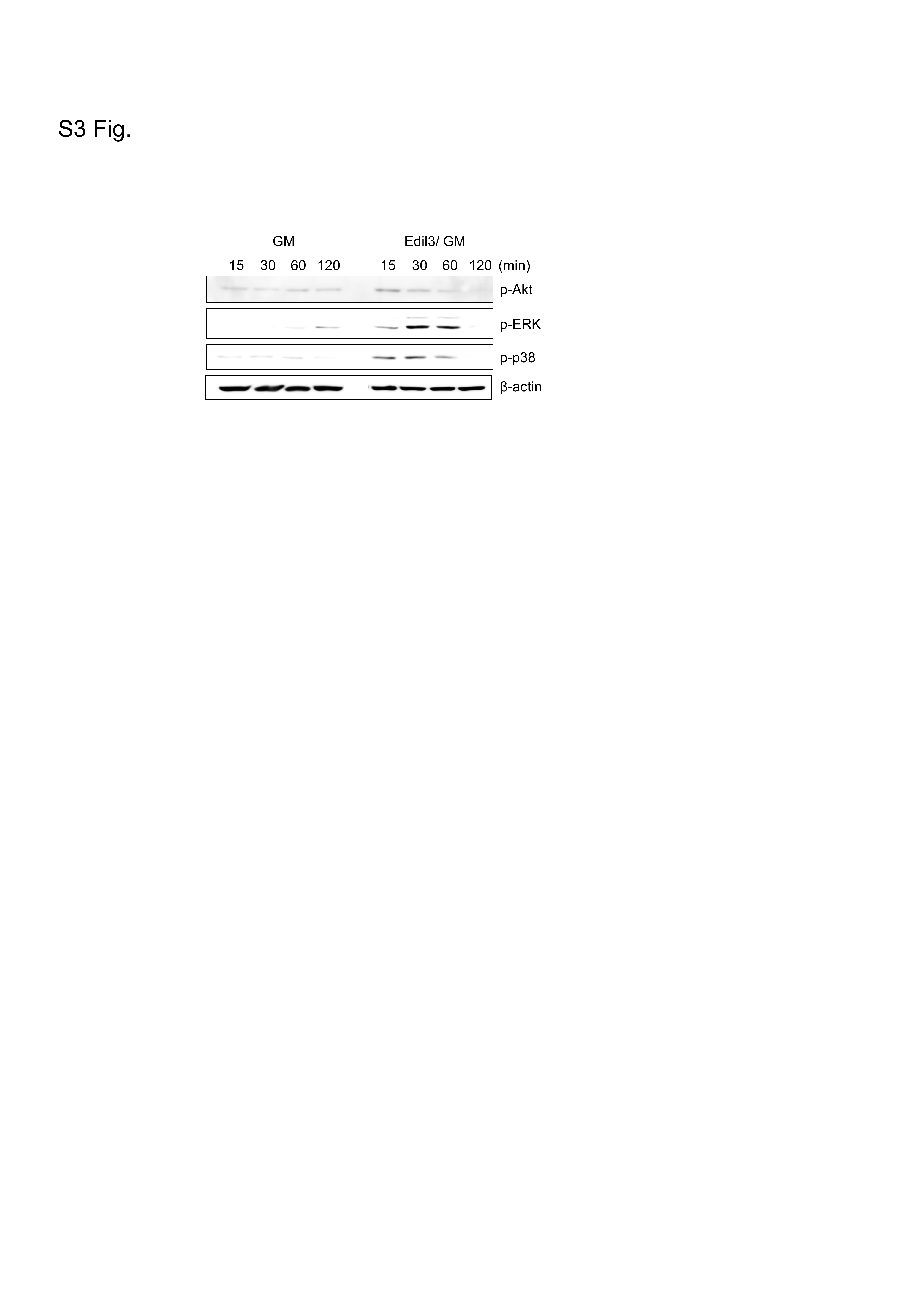

Supplement: S3 Fig — MC3T3-E1 cells were incubated with growth medium for the indicated time period in the absence or presence of Edil3 (200 ng/ml). Cells were harvested and immunoblot analysis was performed using specific antibodies against p-Akt, p-ERK, p-p38, and β-actin. Note that Edil3 treatment increased phosphorylation of these kinases compared to the time-course control without Edil3. (TIF) [file pone.0188749.s003.TIF]
